# Functional role(s) of phagosomal Rab GTPases

**DOI:** 10.4161/sgtp.25604

**Published:** 2013-07-30

**Authors:** Maximiliano Gabriel Gutierrez

**Affiliations:** Division of Mycobacterial Research; Medical Research Council; National Institute for Medical Research; London, UK

**Keywords:** Rab, autophagosome, bacteria, lysosome, phagosome

## Abstract

Rab GTPases are at the central node of the machinery that regulates trafficking of organelles, including phagosomes. Thanks to the unique combination of high quality phagosome purification with highly sensitive proteomic studies, the network of Rab proteins that are dynamically associated with phagosomes during the process of maturation of this organelle is relatively well known. Whereas the phagosomal functions of many of the Rab proteins associated with phagosomes are characterized, the role(s) of most of these trafficking regulators remains to be identified. In some cases, even when the function in the context of phagosome biology is described, phagosomal Rab proteins seem to have similar roles. This review summarizes the current knowledge about the identity and function of phagosomal Rab GTPases, with a particular emphasis on new evidence that clarify these seemingly overlapping Rab functions during phagosome maturation.

## The Process of Phagosome Maturation

The dynamic and complex process of phagosome maturation is the result of multiple interactions between the phagosome and various intracellular compartments.^1^^-^^3^ Biological events happening in the lumen and membrane of phagosomes have a profound impact on the development of an appropriate innate and adaptive immune response.^4^^,^^5^ Compelling evidence from different studies using live cell imaging and proteomic analysis indicates that the classical view of phagosome progression as a linear pathway interacting sequentially with components of the endocytic pathway is a very simplistic view of this process.^6^^,^^7^ These studies have shown that phagosome maturation is a very dynamic process and all kinds of transient and rapid interactions occur simultaneously contributing to the maturation of the phagosome.^7^^,^^8^

## Rab GTPases

Rab proteins have emerged as central regulators of the dynamic process of interactions between phagosomes and intracellular compartments.^2^ Rab GTPases are a large family of small GTPases that regulate intracellular transport.^9^^,^^10^ They operate at different layers of regulation, determining the fusion partners, defining the lipid composition of the membrane via recruitment of specific enzymes, affecting the vesicle motility through molecular motors and modulating vesicular transport through interactions with cytoskeletal components.^9^^,^^10^ Therefore, once the Rab GTPases are localized in membranes, they define the biology of the compartment where they are located. Consequently, a particular Rab network will determine the precise biochemical composition and intracellular behavior of a compartment.^9^

## Multiple Rab Proteins are Associated with Phagosomes

Facilitated by the availability of unique methodologies to purify phagosomes, their composition is relatively well described.^7^^,^^8^^,^^11^^-^^14^ Phagosome purification represents a powerful technique that allows the unambiguous biochemical description of proteins associated with a well-defined organelle.^6^^,^^15^ Thanks to the development and continued improvement of phagosome purification techniques, different groups have studied the recruitment of proteins, including Rab proteins, to phagosomes. These studies clearly showed that the process of phagosome maturation is highly dynamic even at a relatively short time after phagosome formation.^7^^,^^8^ In particular, phagosome purification techniques combined with proteomic studies have clearly shown that multiple Rab GTPases associate to particle-containing phagosomes (Table 1).

**Table:**
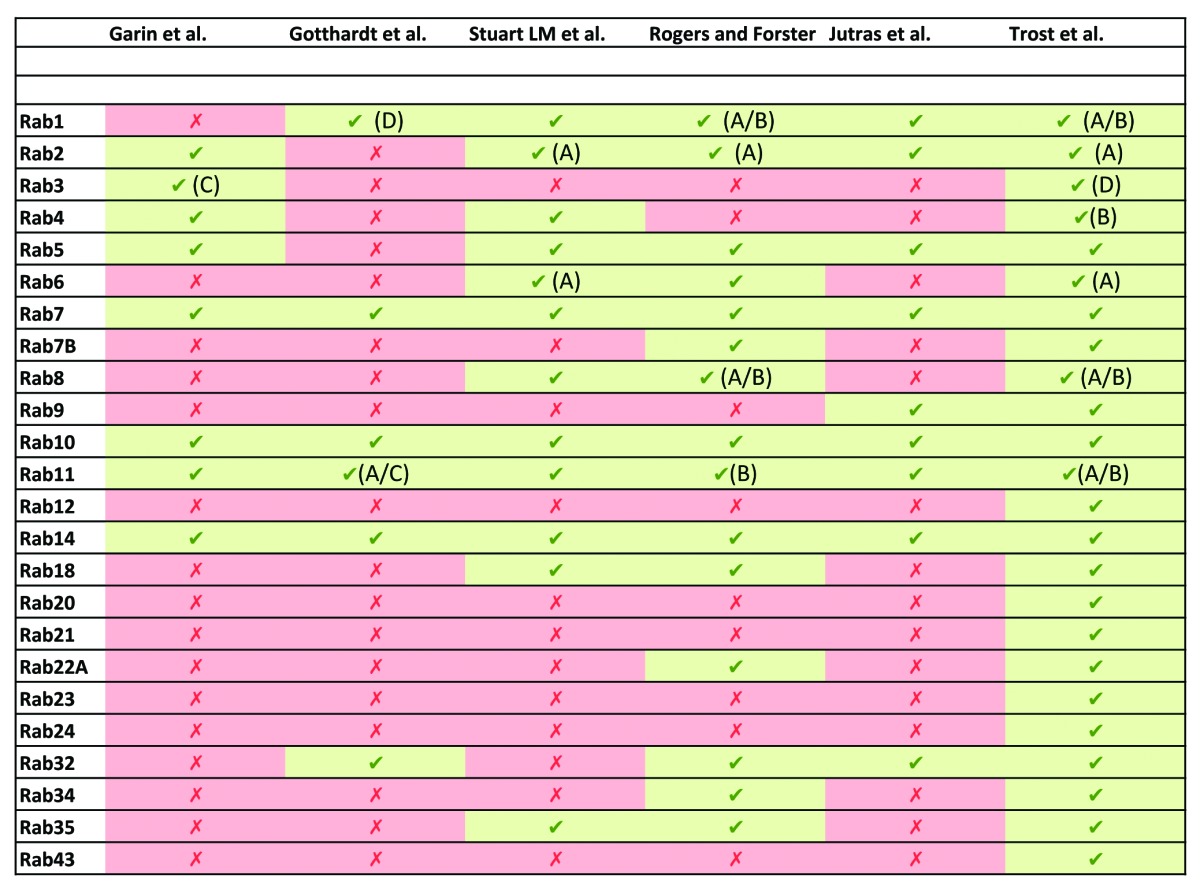
**Table 1.** The phagosomal Rab GTPases. Rab GTPases associated with particle-containing phagosomes in 6 proteomic studies

Proteomes of bacteria-containing phagosomes are not considered here (see text). Letters in brackets refer to the specific isoform detected.

Assuming that the recruitment of specific proteins onto phagosomes is linked to particular functions required in this organelle, specific association of a defined group of Rab proteins would endow the phagosome with a distinct molecular behavior. Although the identities and functions of many phagosomal Rab proteins are relatively well known, their role(s) in the coordination of specific steps within the network of phagosomal Rab GTPases are still poorly defined.

## The Network of Phagosomal Rab GTPases

Although there must be distinctions based on different conditions, protocols of phagosome purification, nature of the ligand used for internalization, methodology used for analysis, age of the analyzed phagosomes, activation status of the cell and obviously the cell type, there are still some common tendencies in the identity of phagosomal Rab proteins (Table 1). Based on the number of times that the Rab proteins were detected under different experimental and technical systems and the level of understanding of the function in phagosomes, phagosomal Rab GTPases can be generally divided into two groups: The first group contains the Rab proteins that are commonly detected in the proteomic studies discussed within this review (Fig. 1). These Rab proteins were identified using phagosomes containing various particles that can be unambiguously isolated and analyzed. The second group of Rab GTPases contains the Rab proteins that are less often identified in the proteomic studies (Fig. 2). This group includes phagosome-associated Rab proteins with unknown or poorly characterized phagosomal functions as well as Rab proteins not commonly identified as being associated with phagosomes but having a well-described phagosomal function.

**Figure:**
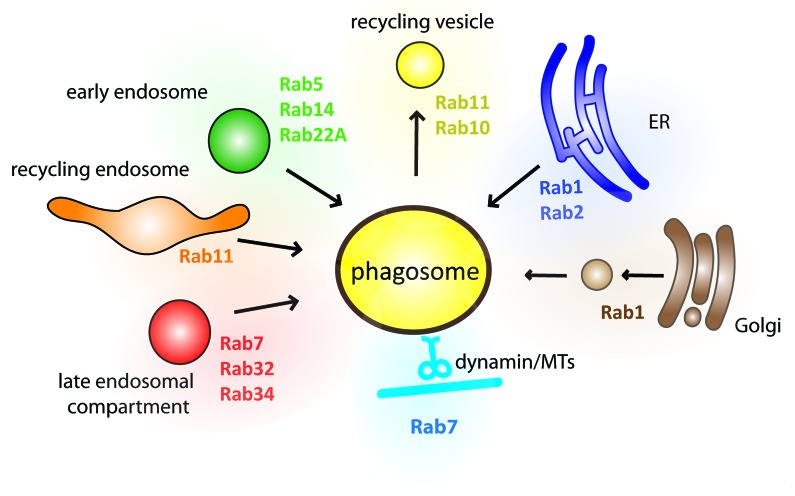
**Figure 1.** The network of phagosomal Rab GTPases: the most common Rab proteins. This model shows the functional link between Rab proteins associated with phagosomes, interactions with different intracellular compartments and/or cytoskeletal components. As discussed in the text, studies have shown that seemingly overlapping functions are in fact more specific. MTs, microtubules; ER, endoplasmic reticulum.

**Figure:**
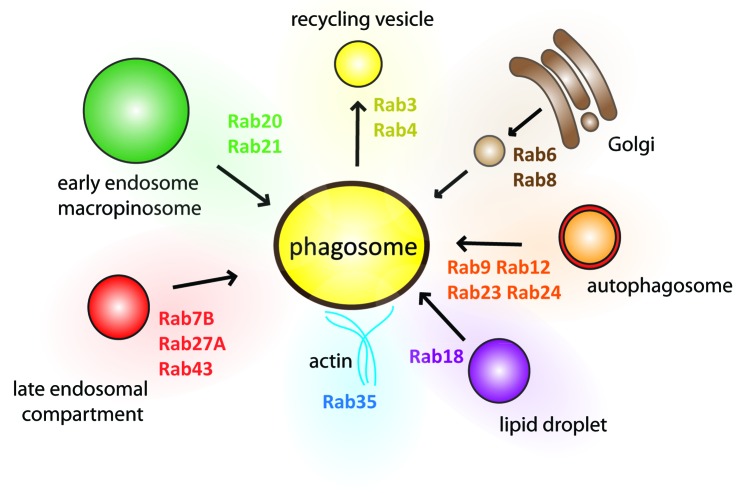
**Figure 2.** The network of phagosomal Rab GTPases: the less common Rab proteins. This model shows the potential functional link between Rab proteins associated with phagosomes and the putative interactions with different intracellular compartments and/or cytoskeletal components.

Other studies have described Rab proteins associated with bacteria-containing phagosomes.^16^^-^^18^ For the sake of simplicity, these bacterial phagosome proteomes are not primarily considered here. To define the composition of bacteria-containing phagosomes is in general more difficult since their composition is very heterogeneous and the risk of contamination with other vesicles is very high. Moreover, intracellular pathogens can manipulate Rab functions increasing the complexity of the Rab proteins associated with different phagosomes, vacuoles, niches etc.^19^ Therefore, as discussed below, the presence of certain proteins in bacterial phagosomes could also reflect active manipulation by the bacteria.

## Rab GTPases Commonly Identified in Phagosomes and with a Relatively Well-Assigned Phagosomal Function (Fig. 1)

### Rab1

This GTPase is mainly related to the transport from the endoplasmic reticulum (ER) to the Golgi complex.^20^ The isoform Rab1A in mammalian and *Drosophila melanogaster* cells and Rab1D in *Dictyostelium discoideum* are recruited into particle-containing phagosomes.^7^^,^^8^^,^^12^^-^^14^ In cells infected with *Legionella pneumophila*, Rab1 is early and efficiently recruited in the *L. pneumophila-*containing vacuole.^21^^,^^22^ It has been demonstrated that *L. pneumophila* uses and manipulates Rab1 to survive within cells.^23^^-^^26^ *Legionella* hijacks the host cell machinery to receive components from the secretory pathway and creates an ER-like niche.^27^ Although the manipulation of the Rab1 function by *Legionella* is the one best characterized for this bacterium, this is not the only pathogen that interacts with Rab1 positive vesicles from the early secretory pathway. Rab1 is also recruited into the acidic *Coxiella burnetti* containing vacuoles and it is required for the growth of this intracellular pathogen.^28^

However, the precise function of Rab1 during phagosome maturation is not well defined. It is possible that Rab1 could potentially be part of a formerly described Golgi to phagosome pathway that could deliver critical components necessary for phagosome maturation.^29^^-^^31^ In another scenario, it could also be possible that Rab1 mediates fusion between the ER and phagosomes^32^^,^^33^ and pathogens use this functional property to create a specific niche that fulfill their own requirements. Alternatively, Rab1 is a potential candidate that could regulate the contribution of the ER to phagocytosis.^34^ Consistent with this notion is the existence of a *Legionella* effector that generates active Rab1, mediating ER-derived vesicles recruitment on the plasma membrane.^35^

### Rab2

Rab2 has been identified in many phagosomal proteomes arguing for an important role of this GTPase in phagosomes.^8^^,^^11^^-^^14^ Similar to Rab1, the small GTPase Rab2 is known to be located on secretory vesicles that traffic between the ER and the Golgi complex.^36^ Mammalian Rab2 controls protein sorting and recycling from pre-Golgi intermediates.^36^ UNC-108 is the homolog of Rab2 in *Caenorhabditis elegans*, which has been shown to regulate apoptotic cell degradation via phagosome maturation in *C. elegans*.^37^^-^^39^ *Brucella* recruits Rab2 into their vacuoles via the specific effector RicA and requires Rab2 for replication.^40^^-^^42^ However, this association may be part of the strategy of *Brucella* to interact with the ER, in analogy to the way *Legionella* utilizes Rab1, since no clear role is known for Rab2 in the phagocytic pathway. The presence of Rab2 on phagosomes could nonetheless highlight the importance of interactions between phagosomes and the early secretory pathway or the ER-Golgi intermediate compartment (ERGIC).^32^

### Rab5

Fusion of phagosomes with endosomes is critical for the process of phagosome maturation.^43^ Thus, it is not surprising that the majority of the proteomic studies identified isoforms of the early endosomal GTPase Rab5 as being associated with phagosomes.^8^^,^^11^^-^^14^ In vitro studies using isolated latex bead phagosomes indicated that Rab5 association with phagosomes is lost during maturation.^44^^,^^45^ Purified latex bead phagosomes fuse with early and late endosomal compartments in vitro in a Rab5-dependent manner.^46^ Rab5 is, together with Rab7, one of the best-characterized Rab proteins not only in the endocytic pathway but also in the context of phagosome maturation (see below). Rab5 is required for phagosome maturation and fusion of phagosomes with early endosomes.^47^^,^^48^ Most of the initial studies were performed using the expression of the dominant negative mutant to evaluate Rab5 loss-of-function. Subsequently, it was confirmed by knocking down Rab5a, that *Listeria*-containing phagosomes had reduced fusion of phagosomes with lysosomes.^49^ Once associated with phagosomes, Rab5 recruits Early Endosomal Antigen-1 (EEA-1). This, together with phosphatidylinositol 3-phosphate (PI3P) generation at the phagosomal membrane, is critical for maturation of latex bead-containing phagosomes.^50^

### Rab7

All the phagosome proteomes considered here have found Rab7 as a phagosomal Rab protein.^7^^,^^8^^,^^11^^-^^14^ Rab7 is required for phagosome maturation in *Dictyostelium*.^51^ In mammalian cells the recruitment and activation of Rab7 alone is insufficient to induce fusion of phagosomes with late endosomes and lysosomes.^47^ From the phagosomal membrane, active Rab7 recruits the effector protein Rab7-interacting lysosomal protein (RILP), which in turn brings the microtubule-associated motor complex dynein-dynactin onto phagosomes. These motors not only drive the phagosomes in the centripetal direction but also induce the extension of phagosomal tubules that contact late endocytic compartments.^52^

Mirroring the mechanism of Rab protein conversion postulated for the endocytic pathway,^53^^-^^55^ evidence suggests that a similar machinery operates in phagosomes.^47^^,^^56^^,^^57^ Moreover, it has been proposed that another endocytic Rab GTPase, Rab22a (see below), regulates the conversion from Rab5 to Rab7 in mycobacterial phagosomes.^58^ However, it is not clear which factors Rab22a recruits into phagosomes that could eventually modulate phagosome conversion. On the other hand, it is not entirely clear if this Rab5 to Rab7 conversion observed in the endocytic pathway also applies to the phagocytic pathway since there are several reports suggesting that Rab7 is present in early phagosomes positive for Rab5.^17^^,^^59^^-^^61^ These data make a compelling case for Rab7 as one of the master regulators of phagosome biology, in particular mediating interactions with the late endocytic/lysosomal compartment.

### Rab10

Rab10 has been consistently identified by proteomic studies as a Rab GTPase associated with phagosomes.^8^^,^^11^^-^^14^ Rab10 is required for endocytosis, recycling, and exocytosis in polarized cells.^62^^,^^63^ On one hand, Rab10 was found to be recruited early into IgG-coated latex beads-containing phagosomes where it regulates LAMP-2 acquisition by phagosomes.^64^ On the other hand, other groups have reported a weak association of Rab10 to phagosomes containing *Staphylococcus aureus*,^61^ *Mycobacterium tuberculosis,*^61^ or *Salmonella*.^65^ These observed differences could be due to the different activation pathways and survival strategies of these pathogens. In terms of function, it has been proposed that Rab10 could be operating via recycling of components required for phagosome maturation.^64^ It is known that phagosome maturation requires the retrieval of certain phagosomal membrane compounds, a process that also involves the GTPase Rab11.^66^ However, the exact nature of these recycled components mediated either by Rab11 (see below) or Rab10 remain to be identified. Based on recent evidence, Rab10 could also be a potential link between the dynamic interactions between phagosomes and the ER.^67^

### Rab11

Isoforms of Rab11 are always present in several particle-containing phagosome proteomes.^7^^,^^8^^,^^11^^,^^13^^,^^14^ Originally, it had been shown that Rab11 participates in the mobilization and recruitment of early endocytic compartments in macrophages to enhance phagocytosis.^68^ Expressed Rab11 is also present in *Salmonella* containing vacuoles.^65^^,^^69^ Evidence suggests that Rab11 is part of the essential machinery that regulates phagosome maturation by increasing the recycling of phagosomal components,^70^ likely via the Rab11/Rab4 effector Rab-coupling protein (RCP).^66^ In fact, in vitro studies have shown that there is a recycling mechanism from the phagosome.^71^ Although intuitively it would be assumed that during multiple fusions with phagosomes, some membrane gets recycled back to maintain a constant compartment size, the precise mechanism and the nature of the recycled components that are required for phagosome maturation are still an incomplete picture. Interestingly, the immune function of Rab11 in macrophages operates via the regulation of fusion between phagosomes and recycling endosomes. Rab11 regulates the delivery of the Toll-like receptor 4 (TLR4) from endocytic recycling compartments into phagosomes. This recruitment is critical for the intracellular signaling of TLR4 from phagosomes containing Gram-negative bacteria. Moreover, Rab11 is required for TRIF-related adaptor molecule (TRAM) recruitment into phagosomes and interferon regulatory transcription factor 3 (IRF3) signaling leading to the secretion of type I interferons.^72^

### Rab14

This GTPase involved in Trans-Golgi Network (TGN) to early endosomes and plasma membrane transport^73^ is present in most of the phagosome proteomes.^7^^,^^8^^,^^11^^-^^14^ The first functional evidence of Rab14 in phagosomes came from studies performed in *D. discoideum*. RabD, a *Dictyostelium* Rab14-related GTPase, localizes in the endo-lysosomal pathway and is an important regulator of homotypic phagosome and endo-lysosome fusion.^74^ In macrophages infected with *Mycobacterium bovis* BCG, Rab14 is actively recruited into phagosomes containing mycobacteria, correlating this association with an impairment in phagosome maturation.^75^ In vitro studies identified Rab14 involvement in fusion between phagosomes and early endosomes, suggesting that Rab14 has a similar function to that of Rab5. In *Salmonella* infected cells, Rab14 is required for intracellular growth of this bacterium.^76^ More information on the functional role of Rab14 in phagosome biology emerged from studies with *S. typhimurium*. The *Salmonella* effector SopB activates Akt1, which in turn phosphorylates AS160, the GTPase activating protein (GAP) of Rab14. This phosphorylation prevents AS160 binding to phagosomal membranes maintaining an active form of Rab14 associated with the phagosome and consequently inhibiting phagosomal maturation.^76^ Altogether, both studies highlight the importance of Rab14 association with *Salmonella* and mycobacteria containing phagosomes to maintain an early phagosomal identity. Interestingly, other pathogens like *Chlamydia* recruit Rab14 and this recruitment is required for enlargement of the Chlamydial replicative niche.^77^ Moreover, in dendritic cells, it has been postulated that Rab14 participates in the recruitment of the insulin-responsive aminopeptidase (IRAP) into phagosomes, regulating the IRAP-dependent cross-presentation pathway in those cells.^78^

In summary, all these studies point out Rab14 as an important Rab protein that regulates interaction of phagosomes with early endocytic compartments. However, the precise role of Rab14 in phagosomes and how the function is different from Rab5 remains to be identified.

### Rab22A

This small GTPase from the endocytic group of GTPases^79^ has been detected in two latex bead phagosome proteomes.^8^^,^^14^ However, expressed Rab22A in macrophages was not detected in latex bead phagosomes by live cell imaging.^58^ Clearly, the comparison between bulk studies such as western blot or Mass Spectrometry vs. single-event, dynamic studies such as live cell imaging is not straightforward and represents an important point to be considered.^58^^,^^64^ Conversely, Rab22A is associated with *M. bovis* BCG phagosomes during the first 50 min.^58^ Remarkably, in phagosomes containing *M. bovis* BCG, Rab22A loss-of-function led to the acquisition of Rab7 into these phagosomes, an event that is partially blocked by the bacteria.^57^^,^^80^ Thus, it appears that the recruitment of Rab22A into phagosomes is an important step of the Rab5 to Rab7 conversion process in *M. bovis* BCG phagosomes. In contrast to latex beads and BCG-containing phagosomes, more than half of phagosomes containing *M. tuberculosis* H37Rv were positive for expressed Rab22A after 10 min in fixed macrophages.^61^ The latter report is in agreement with the localization of Rab22A in early and recycling endosomes^81^ since these early compartments interact with *Mycobacterium avium* phagosomes.^30^^,^^48^ One important aspect to consider in studies that use Rab22A expression is that the overexpression of Rab22A clearly has an effect on the endocytic pathway.^81^ This indicates that the intracellular levels of Rab22A are important and the behavior of the endogenous protein could potentially differ from the overexpressed protein.

Although the molecular components that Rab22A brings into phagosomes to modulate their function remains unknown, it has been described that during infection with *Legionella,* the VipD effector protein prevents the binding of Rab5 and Rab22a to critical downstream effectors such as Rabaptin-5, Rabenosyn-5 and EEA-1 causing a block in lysosomal degradation. Together, this work reveals endosomal trafficking as a host target of *L. pneumophila* and delineates one of the possible underlying molecular mechanisms.^82^

### Rab32

Rab32 was originally reported to regulate mitochondrial dynamics.^83^ Later, it was shown that Rab32 together with Rab38 regulates melanosome biogenesis and likely other lysosome-related organelles.^84^ Rab32 was found to be associated with latex bead phagosomes after 2 h of internalization.^8^ Seto and coworkers reported that expression of the dominant negative mutant of Rab32 impaired the acquisition of the lysosomal enzyme Cathepsin D by latex bead phagosomes.^61^ However, no differences between *M. tuberculosis* H37R virulent (HR7Rv) and H37R avirulent (H37Ra) strains were observed, suggesting that Rab32 recruitment on phagosomes is independent of active mechanisms of bacterial subversion.^61^ However, it is not clear if the blockage occurs at the level of immature or mature Cathepsin D, present in early or late endosomes respectively, since immunolocalization does not allow discrimination between both forms. Moreover, these observations are based on the expression of a dominant negative form of Rab32. Knockdown experiments will be important to confirm the function of Rab32 in mycobacterial survival. In a different experimental setting, Smith and coworkers reported low association of expressed Rab32 to wild-type *Salmonella* phagosomes.^65^ However, an effector protein of *Salmonella* targets Rab32 for degradation allowing survival of this pathogen in mammalian cells.^85^ Moreover, silencing of Rab32 increases *Salmonella* survival in macrophages^85^

In summary, Rab32 has been consistently found as a phagosomal Rab protein and must be important during phagosome maturation.^7^^,^^8^^,^^12^^,^^14^ Though evidence suggests a role in interactions between phagosomes and late endosomes, the precise phagosomal function of Rab32 remains to be identified.

### Rab34

Rab34 was originally described as being associated with phagosomes in proteomic studies.^8^ Rab34 was also described as being transcriptionally dependent of the transcription factor NF-κB during the lysosomal-mediated killing of mycobacteria by macrophages.^86^ These observations strongly suggested a role for this GTPase in phagosome maturation. Moreover, it was shown that Rab34 participated in the delivery of Cathepsin D into phagosomes but the mechanism is unclear.^61^ Recently, Rab34 was shown to have a critical and specific role in phagolysosome biogenesis operating via a size-dependent mechanism of cargo transfer.^87^ Although Rab34 only transiently interacts with phagosomes, knockdown of endogenous Rab34 or overexpression of the Rab34 dominant negative mutant blocks the fusion of phagosomes with lysosomes. Conversely, expression of Rab34 wild type and the constitutively active mutant enhanced phagolysosome biogenesis independently of Rab7.^87^ These studies support a view in which Rab7 and Rab34 perform a largely distinct, but parallel and maybe even complementary functions during phagosome maturation.^87^

## Rab GTPases Less Often Identified in Phagosomes and with Unclear Phagosomal Functions (Fig. 2)

### Rab3

ApRab3, a GTPase 78% identical to the human Rab3C, was originally reported to be associated with symbiosomes and accumulates on the maturing phagosomes in the *Aiptasia pulchella* digestive cells.^88^ In mammals, Rab3 has been largely associated with several intracellular mechanisms of exocytosis.^89^ The isoform Rab3C was originally detected in one of the first phagosomal proteomes^11^ and recently in phagosomes isolated from IFN-γ activated cells.^14^ In a lentivirus-based siRNA screening, Rab3B/C was found to be required for antigen cross-presentation in dendritic cells.^90^ Based on these observations, it is proposed that in dendritic cells, internalized bacteria in phagosomes and Rab3B/C-positive recycling endosomes may constitute an exocytic step of cross-presentation.^90^

### Rab4

The function of this GTPase in phagosome maturation is not known. It has been proposed that RCP present on phagosomes acts as an intermediate between Rab4 and Rab11, regulating recycling events along the phagocytic pathway.^66^ The porin B (PorB) from *Neisseria* induces the early association of Rab4 to latex bead phagosomes.^91^ Expressed Rab4 is present in *Salmonella* containing phagosomes.^65^ Interestingly, the imidazoline-1 receptor (I1R) Nischarin is an effector of both Rab4 and Rab14 and is required for survival and replication of *Salmonella* in host-derived vacuoles.^92^

### Rab6

Rab6, together with Rab33B, coordinate a major intra-Golgi retrograde trafficking pathway but the function of Rab6 in phagosome maturation is not known. This coordination may have parallels with Rab conversion/cascade events that regulate endosomal, phagosomal and exocytic processes.^93^ Moreover, the recruitment of two Golgi-associated Rab proteins, Rab6 and Rab8, on *Salmonella* containing vacuoles was shown to operate via the effector SipC.^94^

### Rab7B

Rab7B has a different function from Rab7^95^^,^^96^ and it has also been identified as being associated with phagosomes.^8^^,^^14^ The function of Rab7B in the context of phagosome maturation is unknown. However, this association is potentially very interesting since it is expressed in macrophages and associated with late endosomes and lysosomes. After LPS treatment, Rab7B is transported to TLR4-positive endosomes leading to TLR4 degradation and signaling. These findings suggest that Rab7B could be a potential negative regulator of TLR4 signaling from the phagosome by promoting the translocation of TLR4 into lysosomes for degradation.^97^

### Rab8

Rab8 function has been linked to diverse processes including cell migration and polarization, neuronal differentiation, and generation of cilia.^98^ Although Rab8 is found in phagosome proteomes, little is known about the function of Rab8 in the context of phagosome maturation. Rab8 has been identified as a component of the *Legionella* containing vacuole from *D. discoideum* suggesting that *Legionella*-containing phagosomes communicate with the secretory pathway.^18^ In the case of *Salmonella*, it has been shown that the effector protein SipC specifically binds and recruits host Syntaxin 6 (Stx6) together with other accessory molecules including Rab6 and Rab8 on *Salmonella*-containing vacuoles.^94^

### Rab9A, Rab12, Rab23 and Rab24

These Rab proteins have been found in phagosome proteomes of macrophages stimulated with IFN-γ.^12^^,^^14^ Interestingly, these small GTPases have all been found to be involved in different steps of autophagy and are present on autophagosomes.^99^^-^^101^

The expression of the dominant-negative mutant of Rab23 inhibits the fusion of *Salmonella*-containing phagosomes with lysosomes.^65^ Moreover, both Rab23 and Rab9A are regulators of autophagy during Group A *Streptococcus* (GAS) infection. Knockdown of Rab9A or Rab23 impairs the killing of intracellular GAS, suggesting that these GTPases play a role in targeting GAS to autophagy and degradation.^102^ Thus, it is likely that these GTPases could represent interesting regulators of interactions between phagosomes and autophagosomes, albeit their role in phagosome maturation remains poorly characterized.

### Rab18

In mammals, Rab18 plays a role in controlling the interactions between lipid droplets and the ER,^103^^,^^104^ in a process regulated by extracellular signals.^105^ Rab18 is involved in lipogenesis as well as in lipolysis, eventually facilitating interaction of lipid droplets with ER membranes and allowing exchange of lipids between these two compartments.^106^ It has been postulated that the maintenance of Rab18 in *Salmonella* vacuoles contributes to the block in transport of phagosomes to lysosomes.^107^ The molecular machinery that regulates lipid body interactions with phagosomes is not well characterized.^108^ Based on the evidence discussed here and given the importance of lipid droplets for intracellular pathogens,^109^ Rab18 could represent a potential link between lipid droplets and phagosomes. However, the functional role of this GTPase in phagosome dynamics remains to be identified.

### Rab20 and Rab21

These GTPases are localized both in early endocytic compartments. Using a dominant negative expression approach, Rab20 has been found to modulate the acquisition of the acidotropic dye lysotracker by latex bead phagosomes.^61^^,^^110^ Expressed Rab20 is associated early to phagosomes.^110^ Altogether, it is likely that Rab20 has a function in phagosome biology although the mechanism is unknown. In the case of Rab21, this GTPase has been found in phagosomes isolated from IFN-γ treated macrophages.^14^ Rab21 function is in the regulation of early endosomal dynamics^111^ and it is associated with macropinosomes in macrophages.^112^ Thus, it is possible that Rab21 regulates early interactions of phagosomes with early endosomes or macropinosomes.

### Rab35

Rab35 regulates actin-dependent phagosome formation by recruiting ACAP2 (ArfGAP with coiled-coil, ankyrin repeats and PH domains 2), which might control actin remodeling and membrane trafficking through ADP-ribosylation factor 6 (Arf6).^113^ Rab35 remains associated with early phagosomes after phagosome formation.^8^^,^^13^^,^^14^ Most of the studies performed to analyze the function of Rab35 indicated that this GTPase regulates assembly of actin filaments during development in Drosophila, filopodia formation^114^^,^^115^ and F-actin generation.^116^ The effect of Rab35 in regulating the localized actin assembly is mediated by the actin-bundling protein fascin.^114^ Altogether, it could be possible that Rab35 contributes to the machinery that assembles actin in phagosomes, a process known to have profound consequences in the fate of phagosomal cargo.^117^^,^^118^

## Conclusions and Outstanding Questions

### The functional network of phagosomal Rab GTPases

Based on the phagosome proteomic data from multiple experimental settings, a group of Rab proteins emerged as critical players of phagosome biology. This group represented in Figure 1, highlights some important phagosomal functions of Rab proteins such as interactions with early and late endocytic compartments, phagosomal recycling and communication with the post-Golgi pathway and the ER. With some exceptions, the studies discussed here argue for overlapping functions of phagosomal Rab proteins. In this way, processes like phagosome acidification, acquisition of early/late endosomal markers or delivery of specific lysosomal enzymes appears to be regulated by multiple Rab proteins. However, increasing evidence indicates that single Rab proteins that become associated with the phagosome can have highly specific function(s).

Interactions of phagosomes with early endocytic compartments are crucial for phagosome maturation.^43^ At least three Rab proteins regulate the early interactions of phagosomes with the endocytic pathway. Clearly, Rab5 is at the center of this regulatory network. In addition, another early endocytic Rab, Rab22A, regulates the switch of Rab5 for Rab7. Therefore, Rab22A represents another layer of regulation, perhaps recruiting specific factors that modulate the phagosomal transition from Rab5 to Rab7. In this context, Rab14 would be a link between early endosome/phagosome interactions and intracellular signals, e.g., AKT1 activation.^76^

Three GTPases, Rab7, Rab32, and Rab34, are reported to regulate fusion of phagosomes with the heterogeneous late endocytic compartment. However, the precise mechanism by which they act is different. Rab7 regulates primarily the transient interactions whereas Rab34 is required for more complete fusion events.^87^ Additionally, Rab7 links phagosomes with molecular motors, microtubule-mediated movement and tubule formation affecting phagosomal transport and fusion.^52^ In this scenario, Rab32 would contribute to the fusion with a subset of vesicles that mainly contain cathepsin D.^61^ Nevertheless, the precise origin and identity of these vesicles positive for both Rab32 and Cathepsin D, remains to be defined.

Regarding the process of phagosomal recycling, Rab11 regulates not only recycling from the phagosomes but also the delivery of TLR4 into phagosomes from recycling endosomes, with important consequences in transcriptional activation and production of cytokines.^72^ In this context, Rab10 could be modulating the recycling of transferrin receptors.^64^

Finally, Rab1 and Rab2 mediate the interactions with the ER, post-Golgi and ERGIC compartments with possible consequences in antigen presentation and phagocytosis. It is important to mention that a pathogen-independent function of these two Rab proteins remain to be described. However, they must have a critical role in phagosome maturation since they are both commonly identified as phagosomal Rab proteins.

### A core vs. accessory/regulated set of phagosomal Rab GTPases?

Proteomic studies revealed a second group of Rab proteins, some of them less well characterized but potentially contributing to specific immune functions of phagosomes (Fig. 2).

Immune cells respond to extracellular stimuli such as cytokines modifying their intracellular trafficking necessities. Thus, the immune modulated function of Rab proteins represents an important level of regulation to consider.^119^ In IFN-γ activated macrophages, the network of Rab GTPases associated with phagosomes changes dramatically.^12^^,^^14^ Many of the phagosomal Rab proteins are similar to those in non-stimulated cells but additionally new Rab proteins are recruited. This group consists of Rab9A, Rab12, Rab20, Rab21, Rab23, Rab24, and Rab43. Hence, it is likely that these IFN-γ dependent Rab proteins might have an important innate immune function in macrophages.

Another step of regulation is represented by specific functions of different cell types such as macrophages and dendritic cells. More specific immune-related pathways could eventually require different Rab GTPases recruitment into phagosomes. This will eventually lead to further specialized functions such as antigen presentation (via MHC I, MHC II, CD1 etc.) and bacterial degradation. A good example of a cell-specific function is the regulation of cross-presentation in dendritic cells by Rab27A. This GTPase regulates the pH of phagosomes in dendritic cells^120^ whereas it enhances phagocytosis in macrophages.^121^ Intriguingly, Rab27A is not detected in any of the proteomes of phagosomes or late endocytic organelles performed in dendritic cells or macrophages.^122^^-^^124^

During internalization of microbial pathogens, there are also pathogen-driven changes in the network of phagosomal Rab GTPases.^16^^,^^61^^,^^65^^,^^125^ One striking example is the highly specific recruitment of Rab29 into *Salmonella enterica* serovar Typhi-containing phagosomes.^126^ Many studies have pointed out that there are not only more but also different Rab proteins in bacteria-containing phagosomes than in particle-containing phagosomes. However, in most of the cases, the functional consequence of this recruitment has not been fully investigated.

Based on the evidence discussed here, it would be possible to reconcile the classical linear model of phagosome maturation (the default pathway) with a more specific e.g., immune regulated pathway (Fig. 3). In this way, during the process of phagosome maturation, a core set of Rab proteins regulates a ‘default’ transport and maturation of phagosomes. This group could be independent of the ligand, immune signals or cell type and regulates critical functions of the phagosome such as interactions with the early and late endocytic compartments. In addition to the core machinery of phagosomal Rab proteins, further recruited Rab proteins could potentially have a specific function in a temporal, immunological or pathogen-driven context during the life of a phagosome. As seen for other small GTPases during endocytosis,^9^ the dynamic network of phagosomal Rab proteins will potentially reflect their ability to perform an specific immune function (Fig. 3).

**Figure:**
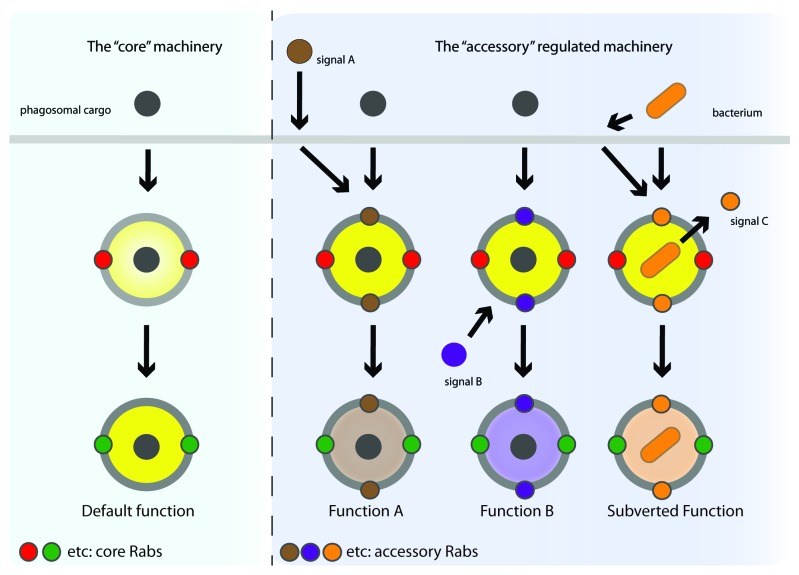
**Figure 3.** The core vs. accessory network of phagosomal Rab GTPases. Proposed model for the observed functional association of multiple Rab GTPases to phagosomes. A group of ‘core’ Rab GTPases regulates the default maturation of the phagosomes as housekeeping Rab proteins (indicated in red and green). Another group, called here accessory Rab GTPases could have a functional impact in a more regulated manner (indicated in brown, purple and orange). First, activation via some intracellular (not depicted) and extracellular signals (shown as ‘signal A’) can change the fate of the phagosome and consequently the function triggered by the origin signal. Alternatively, different intracellular signals e.g., present and/or activated in different cell types (shown as ‘signal B’) can modulate the default maturation. Finally, compelling evidence indicates that pathogen-driven recruitment (either after activation of receptors or secreting bacterial factors, shown as ‘signal C’) of Rab GTPases can change the phagosomal fate facilitating bacterial survival. The model is simplified and does not include well-known situations in which the pathogen-subverted Rab functions are in fact from the core machinery.

The concept of phagosome maturation has a very broad definition and includes biochemical changes in membrane and lumen composition.^3^ The dynamic network of phagosomal Rab GTPases exposes complex functions that converge in phagosome maturation, organizing which components are delivered into the phagosome and which ones are recycled back. In the last few years, studies started to dissect the specific function of individual phagosomal Rab proteins. Nevertheless, the function of the majority of the phagosomal Rab proteins remains poorly characterized.

Deciphering the functional role of every individual phagosomal Rab and how they interact in complex networks during the process of phagosome maturation is critical to understand the link between phagosome biology and the immune response.

## References

[1] Fairn GD, Grinstein S (2012). How nascent phagosomes mature to become phagolysosomes. Trends Immunol.

[2] Flannagan RS, Jaumouillé V, Grinstein S (2012). The cell biology of phagocytosis. Annu Rev Pathol.

[3] Haas A (2007). The phagosome: compartment with a license to kill. Traffic.

[4] Jutras I, Desjardins M (2005). Phagocytosis: at the crossroads of innate and adaptive immunity. Annu Rev Cell Dev Biol.

[5] Stuart LM, Ezekowitz RA (2005). Phagocytosis: elegant complexity. Immunity.

[6] Rogers LD, Foster LJ (2008). Contributions of proteomics to understanding phagosome maturation. Cell Microbiol.

[7] Gotthardt D, Blancheteau V, Bosserhoff A, Ruppert T, Delorenzi M, Soldati T (2006). Proteomics fingerprinting of phagosome maturation and evidence for the role of a Galpha during uptake. Mol Cell Proteomics.

[8] Rogers LD, Foster LJ (2007). The dynamic phagosomal proteome and the contribution of the endoplasmic reticulum. Proc Natl Acad Sci U S A.

[9] Mizuno-Yamasaki E, Rivera-Molina F, Novick P (2012). GTPase networks in membrane traffic. Annu Rev Biochem.

[10] Stenmark H (2009). Rab GTPases as coordinators of vesicle traffic. Nat Rev Mol Cell Biol.

[11] Garin J, Diez R, Kieffer S, Dermine JF, Duclos S, Gagnon E (2001). The phagosome proteome: insight into phagosome functions. J Cell Biol.

[12] Jutras I, Houde M, Currier N, Boulais J, Duclos S, LaBoissière S (2008). Modulation of the phagosome proteome by interferon-gamma. Mol Cell Proteomics.

[13] Stuart LM, Boulais J, Charriere GM, Hennessy EJ, Brunet S, Jutras I (2007). A systems biology analysis of the Drosophila phagosome. Nature.

[14] Trost M, English L, Lemieux S, Courcelles M, Desjardins M, Thibault P (2009). The phagosomal proteome in interferon-gamma-activated macrophages. Immunity.

[15] Griffiths G, Mayorga L (2007). Phagosome proteomes open the way to a better understanding of phagosome function. Genome Biol.

[16] Lee BY, Jethwaney D, Schilling B, Clemens DL, Gibson BW, Horwitz MA (2010). The Mycobacterium bovis bacille Calmette-Guerin phagosome proteome. Mol Cell Proteomics.

[17] Steinhäuser C, Heigl U, Tchikov V, Schwarz J, Gutsmann T, Seeger K (2013). Lipid-labeling facilitates a novel magnetic isolation procedure to characterize pathogen-containing phagosomes. Traffic.

[18] Urwyler S, Nyfeler Y, Ragaz C, Lee H, Mueller LN, Aebersold R (2009). Proteome analysis of Legionella vacuoles purified by magnetic immunoseparation reveals secretory and endosomal GTPases. Traffic.

[19] Stein MP, Müller MP, Wandinger-Ness A (2012). Bacterial pathogens commandeer Rab GTPases to establish intracellular niches. Traffic.

[20] Tisdale EJ, Bourne JR, Khosravi-Far R, Der CJ, Balch WE (1992). GTP-binding mutants of rab1 and rab2 are potent inhibitors of vesicular transport from the endoplasmic reticulum to the Golgi complex. J Cell Biol.

[21] Derré I, Isberg RR (2004). Legionella pneumophila replication vacuole formation involves rapid recruitment of proteins of the early secretory system. Infect Immun.

[22] Kagan JC, Stein MP, Pypaert M, Roy CR (2004). Legionella subvert the functions of Rab1 and Sec22b to create a replicative organelle. J Exp Med.

[23] Ingmundson A, Delprato A, Lambright DG, Roy CR (2007). Legionella pneumophila proteins that regulate Rab1 membrane cycling. Nature.

[24] Murata T, Delprato A, Ingmundson A, Toomre DK, Lambright DG, Roy CR (2006). The Legionella pneumophila effector protein DrrA is a Rab1 guanine nucleotide-exchange factor. Nat Cell Biol.

[25] Müller MP, Peters H, Blümer J, Blankenfeldt W, Goody RS, Itzen A (2010). The Legionella effector protein DrrA AMPylates the membrane traffic regulator Rab1b. Science.

[26] Neunuebel MR, Chen Y, Gaspar AH, Backlund PS, Yergey A, Machner MP (2011). De-AMPylation of the small GTPase Rab1 by the pathogen Legionella pneumophila. Science.

[27] Ninio S, Roy CR (2007). Effector proteins translocated by Legionella pneumophila: strength in numbers. Trends Microbiol.

[28] Campoy EM, Zoppino FC, Colombo MI (2011). The early secretory pathway contributes to the growth of the Coxiella-replicative niche. Infect Immun.

[29] Fratti RA, Chua J, Vergne I, Deretic V (2003). Mycobacterium tuberculosis glycosylated phosphatidylinositol causes phagosome maturation arrest. Proc Natl Acad Sci U S A.

[30] Ullrich HJ, Beatty WL, Russell DG (1999). Direct delivery of procathepsin D to phagosomes: implications for phagosome biogenesis and parasitism by Mycobacterium. Eur J Cell Biol.

[31] Wähe A, Kasmapour B, Schmaderer C, Liebl D, Sandhoff K, Nykjaer A (2010). Golgi-to-phagosome transport of acid sphingomyelinase and prosaposin is mediated by sortilin. J Cell Sci.

[32] Cebrian I, Visentin G, Blanchard N, Jouve M, Bobard A, Moita C (2011). Sec22b regulates phagosomal maturation and antigen crosspresentation by dendritic cells. Cell.

[33] Guermonprez P, Saveanu L, Kleijmeer M, Davoust J, Van Endert P, Amigorena S (2003). ER-phagosome fusion defines an MHC class I cross-presentation compartment in dendritic cells. Nature.

[34] Gagnon E, Duclos S, Rondeau C, Chevet E, Cameron PH, Steele-Mortimer O (2002). Endoplasmic reticulum-mediated phagocytosis is a mechanism of entry into macrophages. Cell.

[35] Arasaki K, Toomre DK, Roy CR (2012). The Legionella pneumophila effector DrrA is sufficient to stimulate SNARE-dependent membrane fusion. Cell Host Microbe.

[36] Tisdale EJ, Jackson MR (1998). Rab2 protein enhances coatomer recruitment to pre-Golgi intermediates. J Biol Chem.

[37] Mangahas PM, Yu X, Miller KG, Zhou Z (2008). The small GTPase Rab2 functions in the removal of apoptotic cells in Caenorhabditis elegans. J Cell Biol.

[38] Guo P, Wang X (2010). Rab GTPases act in sequential steps to regulate phagolysosome formation. Small GTPases.

[39] Guo P, Hu T, Zhang J, Jiang S, Wang X (2010). Sequential action of Caenorhabditis elegans Rab GTPases regulates phagolysosome formation during apoptotic cell degradation. Proc Natl Acad Sci U S A.

[40] de Barsy M, Jamet A, Filopon D, Nicolas C, Laloux G, Rual JF (2011). Identification of a Brucella spp. secreted effector specifically interacting with human small GTPase Rab2. Cell Microbiol.

[41] de Bolle X, Letesson JJ, Gorvel JP (2012). Small GTPases and Brucella entry into the endoplasmic reticulum. Biochem Soc Trans.

[42] Fugier E, Salcedo SP, de Chastellier C, Pophillat M, Muller A, Arce-Gorvel V (2009). The glyceraldehyde-3-phosphate dehydrogenase and the small GTPase Rab 2 are crucial for Brucella replication. PLoS Pathog.

[43] Mayorga LS, Bertini F, Stahl PD (1991). Fusion of newly formed phagosomes with endosomes in intact cells and in a cell-free system. J Biol Chem.

[44] Desjardins M, Huber LA, Parton RG, Griffiths G (1994). Biogenesis of phagolysosomes proceeds through a sequential series of interactions with the endocytic apparatus. J Cell Biol.

[45] Berón W, Colombo MI, Mayorga LS, Stahl PD (1995). In vitro reconstitution of phagosome-endosome fusion: evidence for regulation by heterotrimeric GTPases. Arch Biochem Biophys.

[46] Jahraus A, Tjelle TE, Berg T, Habermann A, Storrie B, Ullrich O (1998). In vitro fusion of phagosomes with different endocytic organelles from J774 macrophages. J Biol Chem.

[47] Vieira OV, Bucci C, Harrison RE, Trimble WS, Lanzetti L, Gruenberg J (2003). Modulation of Rab5 and Rab7 recruitment to phagosomes by phosphatidylinositol 3-kinase. Mol Cell Biol.

[48] Sturgill-Koszycki S, Schaible UE, Russell DG (1996). Mycobacterium-containing phagosomes are accessible to early endosomes and reflect a transitional state in normal phagosome biogenesis. EMBO J.

[49] Alvarez-Dominguez C, Stahl PD (1999). Increased expression of Rab5a correlates directly with accelerated maturation of Listeria monocytogenes phagosomes. J Biol Chem.

[50] Fratti RA, Backer JM, Gruenberg J, Corvera S, Deretic V (2001). Role of phosphatidylinositol 3-kinase and Rab5 effectors in phagosomal biogenesis and mycobacterial phagosome maturation arrest. J Cell Biol.

[51] Rupper A, Grove B, Cardelli J (2001). Rab7 regulates phagosome maturation in Dictyostelium. J Cell Sci.

[52] Harrison RE, Bucci C, Vieira OV, Schroer TA, Grinstein S (2003). Phagosomes fuse with late endosomes and/or lysosomes by extension of membrane protrusions along microtubules: role of Rab7 and RILP. Mol Cell Biol.

[53] Kinchen JM, Ravichandran KS (2010). Identification of two evolutionarily conserved genes regulating processing of engulfed apoptotic cells. Nature.

[54] Rink J, Ghigo E, Kalaidzidis Y, Zerial M (2005). Rab conversion as a mechanism of progression from early to late endosomes. Cell.

[55] Poteryaev D, Datta S, Ackema K, Zerial M, Spang A (2010). Identification of the switch in early-to-late endosome transition. Cell.

[56] Alvarez-Dominguez C, Barbieri AM, Berón W, Wandinger-Ness A, Stahl PD (1996). Phagocytosed live Listeria monocytogenes influences Rab5-regulated in vitro phagosome-endosome fusion. J Biol Chem.

[57] Via LE, Deretic D, Ulmer RJ, Hibler NS, Huber LA, Deretic V (1997). Arrest of mycobacterial phagosome maturation is caused by a block in vesicle fusion between stages controlled by rab5 and rab7. J Biol Chem.

[58] Roberts EA, Chua J, Kyei GB, Deretic V (2006). Higher order Rab programming in phagolysosome biogenesis. J Cell Biol.

[59] Sun J, Deghmane AE, Soualhine H, Hong T, Bucci C, Solodkin A (2007). Mycobacterium bovis BCG disrupts the interaction of Rab7 with RILP contributing to inhibition of phagosome maturation. J Leukoc Biol.

[60] Seto S, Matsumoto S, Ohta I, Tsujimura K, Koide Y (2009). Dissection of Rab7 localization on Mycobacterium tuberculosis phagosome. Biochem Biophys Res Commun.

[61] Seto S, Tsujimura K, Koide Y (2011). Rab GTPases regulating phagosome maturation are differentially recruited to mycobacterial phagosomes. Traffic.

[62] Babbey CM, Ahktar N, Wang E, Chen CC, Grant BD, Dunn KW (2006). Rab10 regulates membrane transport through early endosomes of polarized Madin-Darby canine kidney cells. Mol Biol Cell.

[63] Schuck S, Gerl MJ, Ang A, Manninen A, Keller P, Mellman I (2007). Rab10 is involved in basolateral transport in polarized Madin-Darby canine kidney cells. Traffic.

[64] Cardoso CM, Jordao L, Vieira OV (2010). Rab10 regulates phagosome maturation and its overexpression rescues Mycobacterium-containing phagosomes maturation. Traffic.

[65] Smith AC, Heo WD, Braun V, Jiang X, Macrae C, Casanova JE (2007). A network of Rab GTPases controls phagosome maturation and is modulated by Salmonella enterica serovar Typhimurium. J Cell Biol.

[66] Damiani MT, Pavarotti M, Leiva N, Lindsay AJ, McCaffrey MW, Colombo MI (2004). Rab coupling protein associates with phagosomes and regulates recycling from the phagosomal compartment. Traffic.

[67] English AR, Voeltz GK (2013). Rab10 GTPase regulates ER dynamics and morphology. Nat Cell Biol.

[68] Cox D, Lee DJ, Dale BM, Calafat J, Greenberg SA (2000). A Rab11-containing rapidly recycling compartment in macrophages that promotes phagocytosis. Proc Natl Acad Sci U S A.

[69] Smith AC, Cirulis JT, Casanova JE, Scidmore MA, Brumell JH (2005). Interaction of the Salmonella-containing vacuole with the endocytic recycling system. J Biol Chem.

[70] Leiva N, Pavarotti M, Colombo MI, Damiani MT (2006). Reconstitution of recycling from the phagosomal compartment in streptolysin O-permeabilized macrophages: role of Rab11. Exp Cell Res.

[71] Pitt A, Mayorga LS, Schwartz AL, Stahl PD (1992). Assays for phagosome-endosome fusion and phagosome protein recycling. Methods Enzymol.

[72] Husebye H, Aune MH, Stenvik J, Samstad E, Skjeldal F, Halaas O (2010). The Rab11a GTPase controls Toll-like receptor 4-induced activation of interferon regulatory factor-3 on phagosomes. Immunity.

[73] Junutula JR, De Maziére AM, Peden AA, Ervin KE, Advani RJ, van Dijk SM (2004). Rab14 is involved in membrane trafficking between the Golgi complex and endosomes. Mol Biol Cell.

[74] Harris E, Cardelli J (2002). RabD, a Dictyostelium Rab14-related GTPase, regulates phagocytosis and homotypic phagosome and lysosome fusion. J Cell Sci.

[75] Kyei GB, Vergne I, Chua J, Roberts E, Harris J, Junutula JR (2006). Rab14 is critical for maintenance of Mycobacterium tuberculosis phagosome maturation arrest. EMBO J.

[76] Kuijl C, Savage ND, Marsman M, Tuin AW, Janssen L, Egan DA (2007). Intracellular bacterial growth is controlled by a kinase network around PKB/AKT1. Nature.

[77] Capmany A, Damiani MT (2010). Chlamydia trachomatis intercepts Golgi-derived sphingolipids through a Rab14-mediated transport required for bacterial development and replication. PLoS One.

[78] Weimershaus M, Maschalidi S, Sepulveda F, Manoury B, van Endert P, Saveanu L (2012). Conventional dendritic cells require IRAP-Rab14 endosomes for efficient cross-presentation. J Immunol.

[79] Pereira-Leal JB, Seabra MC (2001). Evolution of the Rab family of small GTP-binding proteins. J Mol Biol.

[80] Gutierrez MG, Master SS, Singh SB, Taylor GA, Colombo MI, Deretic V (2004). Autophagy is a defense mechanism inhibiting BCG and Mycobacterium tuberculosis survival in infected macrophages. Cell.

[81] Mesa R, Magadán J, Barbieri A, López C, Stahl PD, Mayorga LS (2005). Overexpression of Rab22a hampers the transport between endosomes and the Golgi apparatus. Exp Cell Res.

[82] Ku B, Lee KH, Park WS, Yang CS, Ge J, Lee SG (2012). VipD of Legionella pneumophila targets activated Rab5 and Rab22 to interfere with endosomal trafficking in macrophages. PLoS Pathog.

[83] Alto NM, Soderling J, Scott JD (2002). Rab32 is an A-kinase anchoring protein and participates in mitochondrial dynamics. J Cell Biol.

[84] Wasmeier C, Romao M, Plowright L, Bennett DC, Raposo G, Seabra MC (2006). Rab38 and Rab32 control post-Golgi trafficking of melanogenic enzymes. J Cell Biol.

[85] Spanò S, Galán JEA (2012). A Rab32-dependent pathway contributes to Salmonella typhi host restriction. Science.

[86] Gutierrez MG, Mishra BB, Jordao L, Elliott E, Anes E, Griffiths G (2008). NF-kappa B activation controls phagolysosome fusion-mediated killing of mycobacteria by macrophages. J Immunol.

[87] Kasmapour B, Gronow A, Bleck CK, Hong W, Gutierrez MG (2012). Size-dependent mechanism of cargo sorting during lysosome-phagosome fusion is controlled by Rab34. Proc Natl Acad Sci U S A.

[88] Hong MC, Huang YS, Lin WW, Fang LS, Chen MC (2009). ApRab3, a biosynthetic Rab protein, accumulates on the maturing phagosomes and symbiosomes in the tropical sea anemone, Aiptasia pulchella. Comp Biochem Physiol B Biochem Mol Biol.

[89] Fischer von Mollard G, Stahl B, Li C, Südhof TC, Jahn R (1994). Rab proteins in regulated exocytosis. Trends Biochem Sci.

[90] Zou L, Zhou J, Zhang J, Li J, Liu N, Chai L (2009). The GTPase Rab3b/3c-positive recycling vesicles are involved in cross-presentation in dendritic cells. Proc Natl Acad Sci U S A.

[91] Mosleh IM, Huber LA, Steinlein P, Pasquali C, Günther D, Meyer TF (1998). Neisseria gonorrhoeae porin modulates phagosome maturation. J Biol Chem.

[92] Kuijl C, Pilli M, Alahari SK, Janssen H, Khoo PS, Ervin KE (2013). Rac and Rab GTPases dual effector Nischarin regulates vesicle maturation to facilitate survival of intracellular bacteria. EMBO J.

[93] Starr T, Sun Y, Wilkins N, Storrie B (2010). Rab33b and Rab6 are functionally overlapping regulators of Golgi homeostasis and trafficking. Traffic.

[94] Madan R, Rastogi R, Parashuraman S, Mukhopadhyay A (2012). Salmonella acquires lysosome-associated membrane protein 1 (LAMP1) on phagosomes from Golgi via SipC protein-mediated recruitment of host Syntaxin6. J Biol Chem.

[95] Yang M, Chen T, Han C, Li N, Wan T, Cao X (2004). Rab7b, a novel lysosome-associated small GTPase, is involved in monocytic differentiation of human acute promyelocytic leukemia cells. Biochem Biophys Res Commun.

[96] Progida C, Cogli L, Piro F, De Luca A, Bakke O, Bucci C (2010). Rab7b controls trafficking from endosomes to the TGN. J Cell Sci.

[97] Wang Y, Chen T, Han C, He D, Liu H, An H (2007). Lysosome-associated small Rab GTPase Rab7b negatively regulates TLR4 signaling in macrophages by promoting lysosomal degradation of TLR4. Blood.

[98] Peränen J (2011). Rab8 GTPase as a regulator of cell shape. Cytoskeleton (Hoboken).

[99] Nishida Y, Arakawa S, Fujitani K, Yamaguchi H, Mizuta T, Kanaseki T (2009). Discovery of Atg5/Atg7-independent alternative macroautophagy. Nature.

[100] Matsui T, Fukuda M (2013). Rab12 regulates mTORC1 activity and autophagy through controlling the degradation of amino-acid transporter PAT4. EMBO Rep.

[101] Munafó DB, Colombo MI (2002). Induction of autophagy causes dramatic changes in the subcellular distribution of GFP-Rab24. Traffic.

[102] Nozawa T, Aikawa C, Goda A, Maruyama F, Hamada S, Nakagawa I (2012). The small GTPases Rab9A and Rab23 function at distinct steps in autophagy during Group A Streptococcus infection. Cell Microbiol.

[103] Ozeki S, Cheng J, Tauchi-Sato K, Hatano N, Taniguchi H, Fujimoto T (2005). Rab18 localizes to lipid droplets and induces their close apposition to the endoplasmic reticulum-derived membrane. J Cell Sci.

[104] Martin S, Parton RG (2008). Characterization of Rab18, a lipid droplet-associated small GTPase. Methods Enzymol.

[105] Martin S, Driessen K, Nixon SJ, Zerial M, Parton RG (2005). Regulated localization of Rab18 to lipid droplets: effects of lipolytic stimulation and inhibition of lipid droplet catabolism. J Biol Chem.

[106] Pulido MR, Diaz-Ruiz A, Jiménez-Gómez Y, Garcia-Navarro S, Gracia-Navarro F, Tinahones F (2011). Rab18 dynamics in adipocytes in relation to lipogenesis, lipolysis and obesity. PLoS One.

[107] Hashim S, Mukherjee K, Raje M, Basu SK, Mukhopadhyay A (2000). Live Salmonella modulate expression of Rab proteins to persist in a specialized compartment and escape transport to lysosomes. J Biol Chem.

[108] Melo RC, Dvorak AM (2012). Lipid body-phagosome interaction in macrophages during infectious diseases: host defense or pathogen survival strategy?. PLoS Pathog.

[109] Saka HA, Valdivia R (2012). Emerging roles for lipid droplets in immunity and host-pathogen interactions. Annu Rev Cell Dev Biol.

[110] Egami Y, Araki N (2012). Rab20 regulates phagosome maturation in RAW264 macrophages during Fc gamma receptor-mediated phagocytosis. PLoS One.

[111] Simpson JC, Griffiths G, Wessling-Resnick M, Fransen JA, Bennett H, Jones AT (2004). A role for the small GTPase Rab21 in the early endocytic pathway. J Cell Sci.

[112] Egami Y, Araki N (2009). Dynamic changes in the spatiotemporal localization of Rab21 in live RAW264 cells during macropinocytosis. PLoS One.

[113] Egami Y, Fukuda M, Araki N (2011). Rab35 regulates phagosome formation through recruitment of ACAP2 in macrophages during FcγR-mediated phagocytosis. J Cell Sci.

[114] Zhang J, Fonovic M, Suyama K, Bogyo M, Scott MP (2009). Rab35 controls actin bundling by recruiting fascin as an effector protein. Science.

[115] Chua CE, Lim YS, Tang BL (2010). Rab35--a vesicular traffic-regulating small GTPase with actin modulating roles. FEBS Lett.

[116] Dambournet D, Machicoane M, Chesneau L, Sachse M, Rocancourt M, El Marjou A (2011). Rab35 GTPase and OCRL phosphatase remodel lipids and F-actin for successful cytokinesis. Nat Cell Biol.

[117] Marion S, Hoffmann E, Holzer D, Le Clainche C, Martin M, Sachse M (2011). Ezrin promotes actin assembly at the phagosome membrane and regulates phago-lysosomal fusion. Traffic.

[118] Liebl D, Griffiths G (2009). Transient assembly of F-actin by phagosomes delays phagosome fusion with lysosomes in cargo-overloaded macrophages. J Cell Sci.

[119] Pei G, Bronietzki M, Gutierrez MG (2012). Immune regulation of Rab proteins expression and intracellular transport. J Leukoc Biol.

[120] Jancic C, Savina A, Wasmeier C, Tolmachova T, El-Benna J, Dang PM (2007). Rab27a regulates phagosomal pH and NADPH oxidase recruitment to dendritic cell phagosomes. Nat Cell Biol.

[121] Yokoyama K, Kaji H, He J, Tanaka C, Hazama R, Kamigaki T (2011). Rab27a negatively regulates phagocytosis by prolongation of the actin-coating stage around phagosomes. J Biol Chem.

[122] Buschow SI, Lasonder E, Szklarczyk R, Oud MM, de Vries IJ, Figdor CG (2012). Unraveling the human dendritic cell phagosome proteome by organellar enrichment ranking. J Proteomics.

[123] Duclos S, Clavarino G, Rousserie G, Goyette G, Boulais J, Camossetto V (2011). The endosomal proteome of macrophage and dendritic cells. Proteomics.

[124] Li Q, Singh CR, Ma S, Price ND, Jagannath C (2011). Label-free proteomics and systems biology analysis of mycobacterial phagosomes in dendritic cells and macrophages. J Proteome Res.

[125] Urwyler S, Brombacher E, Hilbi H (2009). Endosomal and secretory markers of the Legionella-containing vacuole. Commun Integr Biol.

[126] Spanò S, Liu X, Galán JE (2011). Proteolytic targeting of Rab29 by an effector protein distinguishes the intracellular compartments of human-adapted and broad-host Salmonella. Proc Natl Acad Sci U S A.

